# Can the Neuromuscular Performance of Young Athletes Be Influenced by Hormone Levels and Different Stages of Puberty?

**DOI:** 10.3390/ijerph17165637

**Published:** 2020-08-05

**Authors:** Paulo Francisco de Almeida-Neto, Dihogo Gama de Matos, Vanessa Carla Monteiro Pinto, Paulo Moreira Silva Dantas, Tatianny de Macêdo Cesário, Luíz Felipe da Silva, Alexandre Bulhões-Correia, Felipe José Aidar, Breno Guilherme de Araújo Tinôco Cabral

**Affiliations:** 1 Department of Physical Education, Federal University of Rio Grande do Norte, Natal 59078-970, Brazil; vanecmpinto@gmail.com (V.C.M.P.); pgdantas@icloud.com (P.M.S.D.); tatiannymc@gmail.com (T.d.M.C.); remoesporterowing@hotmail.com (L.F.d.S.); alexandrebulhoescorreia@gmail.com (A.B.-C.); brenotcabral@gmail.com (B.G.d.A.T.C.); 2 Group of Studies and Research of Performance, Sport, Healt and Paralympic Sports GEPEPS, the Federal University of Sergipe—UFS, São Cristovão 49100-000, Brazil; dihogogmc@hotmail.com (D.G.d.M.); fjaidar@gmail.com (F.J.A.); 3 Department of Physical Education, Federal University of Sergipe—UFS, São Cristovão 49100-000, Brazil; 4 Graduate Program in Master’s Level at Department of Physical Education, Federal University of Sergipe—UFS, São Cristovão 49100-000, Brazil; 5 Program of Physiological Science, Federal University of Sergipe—UFS, São Cristovão 49100-000, Brazil

**Keywords:** testosterone, estradiol, sports performance, young athletes, puberty

## Abstract

Background: Endocrine mechanisms can be a determining factor in the neuromuscular performance of young athletes. Objective: The objective of the present study was to relate maturational and hormonal markers to neuromuscular performance, as well as to verify whether young athletes with different testosterone levels show differences in muscle strength. Methods: The sample consisted of 37 young male Brazilian athletes (11.3 ± 0.94 years) who were members of a sports initiation project. Hormonal markers were analyzed biochemically by blood samples, and maturation markers by mathematical models based on anthropometry. Body composition was verified by tetrapolar bioimpedance. The performance of upper and lower limb strength and body speed were analyzed. Results: Hormonal and maturational markers were related to neuromuscular performance (*p* < 0.05). Young people with higher testosterone levels showed higher muscle strength (*p* < 0.05). Artificial neural networks showed that testosterone predicted the performance of upper limbs by 49%, and maturation by 60%. Maturation foreshadowed the performance of lower limbs by 30.3%. Conclusion: Biological maturation and hormonal levels can be related to neuromuscular performance, and young people with higher testosterone levels show superior muscle strength in relation to the others.

## 1. Introduction

The quality of neuromuscular performance, especially of the upper and lower limbs, has been identified as a determinant for physical health and success in several sports skills [1,2]. However, several biological and environmental factors can interfere with the neuromuscular quality of skills that require the use of motor coordination and muscle strength [3,4,5]. At the biological level, we can highlight the puberty process, which is the triggering of improvements in neurological, muscular, skeletal, and endocrine systems [6]. Among the environmental factors, we can highlight the level of physical activity, nutrition and quality of sleep, which are extremely important for the quality of the biological maturation process [7,8].

The interaction between environmental and biological determinants can influence the pace of puberty process, that is, individuals born on the same chronological date may have different biological ages due to these interactions [9]. In this sense, puberty can be classified as early, synchronized or late; these different levels are determined according to the timing of the chronological and biological ages [10]. Each stage of puberty has different characteristics in relation to biological maturation; the endocrine system receives different stimuli, and hormonal levels stand out as one of the main differences between the different levels of puberty [11,12]. Subjects with precocious puberty generally have hormonal levels higher than those of late and synchronized puberty [13,14].

The maturation process resulting from adolescence is strongly governed by reactions from a range of hormones, which affect the maturation rhythm of young people in favor of the increase in sex hormones [15]. Sex hormones, such as estradiol, testosterone, and growth hormone, have extremely important functions in human biological development, which can mainly affect morphological structure, skeletal growth, and muscle strength levels [9,10,11,12,13,14,15,16,17]. In this context, among neuromuscular parameters, muscle strength and body speed are two of the most essential for body functionality in relation to physical health, and for the development of specific skills related to sports performance [2,3,4,5,6,7,8,9,10,11,12,13,14,15,16,17,18,19].

In female subjects, as puberty advances, the levels of steroid hormones tend to increase, pointing to a significant relationship with the levels of muscle strength in the upper and lower limbs [14]. In addition, the secretion of steroid hormones during puberty generates changes in the physical profiles and secondary sexual characteristics of subjects of both sexes [20,21]. At the endocrine level, the stages of advanced puberty are characterized by a predominance of sex hormones in the body [14,15]. However, young people from the same maturation stage may have different levels of hormones in the body [22]. Maturation cannot be the only determinant of neuromuscular characteristics in the pediatric population; analyses of hormone thresholds are necessary to obtain a parameter with a central tendency that serves as a reference point to group the subjects [22,23,24].

The stages of puberty relate to physical abilities. This aspect is widely addressed in sports with the aim of obtaining information about the process of detecting and guiding young talents [1]. In addition, hormonal markers also point to relationships with physical abilities [14]. In this context, both themes should be observed together so that more accurate results can be obtained in relation to factors related to neuromuscular performance in young athletes, especially when observing that motor performance can provide crucial information in the talent selection process in sport.

In this context, in scientific research linear analyzes are interesting to discriminate the importance of variables in relation to performance in sport [14]. Moreover, non-linear patterns can be significant in relation to the characteristics analyzed [14]. In this sense, linear (i.e., regression) and non-linear (i.e., artificial neural networks) analyses have been shown to be effective in providing specific data, in relation to the muscular strength of young sports initiation athletes [14].

Given the assumptions, the present study hypothesized that: (1) young males with higher concentrations of testosterone and with more advanced pubertal stage may be superior in neuromuscular performance when compared with young people with lower concentrations of testosterone and with later pubertal stage; (2) biological maturation and hormonal markers may be related to the neuromuscular performance of young males. Therefore, the aim of the present study was to relate maturational and hormonal markers to neuromuscular performance, as well as to verify whether young athletes with different testosterone levels show differences in muscle strength.

## 2. Methods

### 2.1. Sample

The study was observational with a cross-sectional design. The sample consisted of 37 male adolescent athletes (age of 11.3 ± 0.94 years) who were members of a sports initiation project in the city of Natal, Brazil. The sample size for this research was established a priori, based on a previous study [13], and through an effect size of 0.66 and an α < 0.05 with a β = 0.80. As inclusion criteria, volunteers were between 10 and 12 years old, male and had no clinically diagnosed hormonal dysfunction. All participants who took food or hormonal supplements or who performed vigorous activities in the last 24 h before the exams were excluded.

### 2.2. Ethics

This research was analyzed and approved by the Ethics and Research Committee-CEP of the Federal University of Rio Grande do Norte (Opinion: 1249937), according to resolution 466/12 of the National Health Council on 12/12/2012, while strictly respecting the ethical principles contained in the Declaration of Helsinki. In addition, the present study complies with all items on the STROBE checklist for observational studies (i.e., checklist to strengthen the reporting of observational studies in epidemiology) [25].

## 3. Procedures

**Figure 1:**
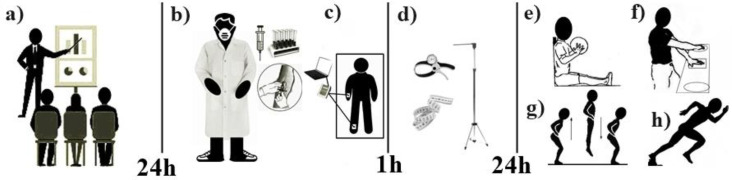
Study design. a = Study information for volunteers and their respective guardians. b = Biochemical tests. c = Body composition test. d = Collecting anthropometric measurements. e = Upper limb power test. f = Upper limb speed test. g = Lower limb power test. h = Body speed with change of direction test.

The sample participants were accompanied by a team of nursing and physical education professionals previously trained and qualified by the Federal University of Rio Grande do Norte to carry out the data collection. Thus, the sample came to the laboratory on three occasions. The first visit was used to inform the volunteers and their respective guardians about the research objectives and the methodology adopted in the study. On the second visit, which took place 24 h after the first, after the volunteers and their respective guardians signed the terms of free and informed consent, blood samples were taken from the fasting individuals for biochemical tests referring to hormonal markers. Next, the body composition test was applied using a tetrapolarbioimpedance scale, and volunteers were offered a snack after an hour-long break, anthropometric measurements were taken. On the third visit, again after 24 h, arm and leg strength and body speed tests were performed, in that order. During the second and third visits, the volunteers were instructed to suspend vigorous activities in the 24 h prior to data collection (Figure 1). It should be noted that during the evaluations the sample and its guardians did not have access to any of the results during the tests performed.

## 4. Body Composition Assessment

The body composition examination was performed using a tetrapolar bioimpedance equipment model BIA1010 with high precision ((Resistance: Range: 0–1000 Ohms; FS: 1000 Ohms; Resolution: 0.1 Ohm; Accuracy = 0.5% FS); (Reactance: Range: 0–1000 Ohms; FS: 1000 Ohms; Resolution: 0.1 Ohm; Accuracy = 1% FS))(Sanny^®^, São Paulo, Brazil), where the evaluated subject was placed in the supine position on a stretcher isolated from electric conductors, and remained at rest for 10 min without wearing any metallic object (i.e., earrings, bracelets, piercings, etc.). Subsequently, emitting electrodes were placed on the surface of the hand and the right foot, close to the joints of the metacarpal and metatarsal phalanges, respectively. The receiving electrodes were placed at the midpoint between the distal prominences of the radius and the ulna of the right wrist, and between the medial and lateral malleolus of the right ankle. Subsequently, pediatric algorithms were selected in the equipment and after the procedures, the evaluator activated the bioimpedance, and the equipment recorded the body composition values through a portable computer attached to the device.

## 5. Anthropometric Assessments

The anthropometric evaluation was carried out through body mass, height, perimeters, bone diameters and skin folds. All assessments were based on the ISAK protocol (International Society of the Advancement of Kinanthropometry) [26]. Body mass was measured using a digital scale (Filizola^®^ São Paulo, Brazil; with a capacity of up to 150 kg and a variation of 0.10 kg); height was measured with a stadiometer (Sanny^®^, São Paulo, Brazil; accurate to 0.1 mm); skinfolds were measured using a scientific adipometer (Harpenden^®^, Londres, Inglaterra; John Bull Indicators Ltd.; accurate to 0.1 mm); perimetry was measured using an anthropometric tape (Sanny^®^, São Paulo, Brazil); an anthropometric ruler (Sanny^®^, São Paulo, Brazil) measured the length of the tibia and femur; and a caliper (Sanny^®^, São Paulo, Brazil) was used for bone diameters of the humerus and femur.

## 6. Biological Maturation Analysis

Biological maturation was analyzed using three distinct parameters: skeletal maturation, which refers to the growth of the human skeleton and the closure of the main bone epiphyzes; sexual maturation, which is associated with secondary sexual characteristics (i.e., pubic hair and genital growth); and somatic maturation, which is linked to the morphology of biological tissues—epithelial, connective, muscle and nervous tissues). Skeletal maturation was analyzed through bone age, which was verified by the equation proposed by Cabral et al. [27]. The equation is highly reliable compared with the gold standard hand and wrist X-ray method (r = 0.868; r² = 0.754; *p* < 0.05). The mathematical model consists of the formula:

Bone age = −11.620 + 7.004 × (height (cm)) + 1.226 × (Dsexo) + 0.749 × (age)−0.068 × (triceps skinfold (mm)) + 0.214 × (corrected arm circumference (cm))−0.588 × (humerus diameter (cm)) + 0.388 × (femoral diameter (cm)).(1)

For the male Dsexo = 0. For the female Dsexo = 1.

To determine maturation stage, the result of skeletal maturation must be compared with the chronological age and when the subject presents values below the chronological age the individual is classified as late, when the values are similar they are classified as synchronous, and when bone age value exceeds the value of chronological age the individual is classified as accelerated [1].

Sexual maturation was verified by the mathematical model proposed by Medeiros et al. [28]. The equation has a strong validation coefficient with the sexual maturation exam performed using a medical exam in a specific office (Kappa validation coefficient = 0.840; *p* < 0.05). The formula is as follows:

Male sexual maturation = (0.49436 × age) + (10.74526 × trunk height (cm)) + 0.11583 × acromion length (cm)) − (0.1394 × tibial length (cm)) − (0.2808 × femur length (cm)) + (0.5963 × forearm circumference (cm)) + (0.22397 × neck circumference (cm)) − (0.5155 × waist circumference (cm)) − 19.69139.(2)

Based on the score resulting from the equation, the stages of sexual maturity are estimated according to the following discriminant score (DS) values: prepubertal stage 1 (DS ≤ −1.67491), pubertal stage 2 (−1.67491 ≤ DS ≤ −0.79575), stage 3 pubertal (−0.79575 ≤ DS ≤ 0.27226), stage 4 post-pubertal (0.27226 ≤ DS ≤ 2.21446) and stage 5 post-pubertal (DS ≥ 2.21446).

Somatic maturation was verified by the equation proposed by Mirwald et al. [29]. The equation has a strong validation with the longitudinal monitoring of maturation through bone mineral accumulation (r = 0.959; r² = 0.920; *p* < 0.05) and is as follows:

Peak of speed growth (PSG) in male sex = −9.236 + [0.0002708 × (leg length (cm) × trunk height (cm))] + [−0.001663 × (age × leg length (cm))] + [0.007216 × (age × trunk height (cm))] + [0.02292 × (weight (kg)/ stature (cm)) × 100](3)

Based on the final values of the equation results, in relation to chronological age, subjects can be classified into three stages of somatic maturation: (1) Pre-PSG (PSG < −1); (2) During PSG (PSG ≥ −1 or PSG ≤ + 1); and (3) Post-PSG (PSG > +1).

## 7. Neuromuscular Performance Analysis

### 7.1. Upper Limb Power

In this study, the upper limb power (ULP) was analyzed using the medicine ball test, previously validated in Brazilian children [30]. Participants were asked to sit with their backs against a wall and their knees extended. At the evaluator’s signal, a 2-kg medicine ball (Ax Sports^®^, Tangará, Brazil) was positioned at the height of the sternum and they were asked to throw it horizontally using both hands. The use of trunk movement was not allowed. The test was performed consecutively three times, interspersed with a passive recovery period of 3 min. The best attempt measurement was considered for the analysis.

### 7.2. Lower Limb Power

Lower limb power was assessed by a squat jump (SJ) using a jump mat (Cefise^®^, Brazil) connected to the Jump Test Pro 2.10 software [31]. It is noteworthy that the test used is effective and feasible in Brazilian children and adolescents [13,14]. To reduce errors while executing the protocol, participants were familiarized with the test. Starting from an orthostatic position, held for 3 s, with the knees flexed at approximately 90º and the hands fixed on the waist, the participants were instructed to perform the jump as high as possible. Three attempts were made, interspersed with 40 s of passive recovery, and the best attempt measurement was included for data analysis.

### 7.3. Upper Limb Speed

To assess upper limb speed (ULS), participants performed the tapping test, previously validated in children and adolescents, in which the participant uses the dominant hand in the shortest possible time [32]. The test is performed with the participant in front of a table of adjustable height to the hip level so that the non-dominant hand is positioned in a central rectangle drawn on the table. The participants then touch the disks drawn on the sides of the table with their dominant hand. In this study, each participant performed 25 cycles between one side disk and another in the shortest possible time.

### 7.4. Body Speed with Change of Direction

The body speed test with change of direction (BScD) was conducted according to the recommendations of Nimphius et al. [33]. Two vertical lines were drawn on the ground with a distance of 10 m between them and the central point was positioned in the middle of the 5-m distance marked on the ground with the drawing of a circle. This helped the participants run as fast as they could from the circle, touch the left line, change direction, touch the right line, and return to the central circle, immediately starting the same procedure again three times in a row. This protocol was performed in two attempts, interspersed with a 3-min passive recovery time, and the best attempt measurement was considered for data analysis.

### 7.5. Biochemical Analyses of Hormone Levels

Peripheral blood samples (10 mL) from the antecubital vein were obtained from participants to analyze hormone levels. The blood sample tubes were centrifuged with a clot activator at 6000 rpm for 10 min, to obtain 0.5 mL of serum. The serum samples were kept on ice at −20 °C and monitored directly (the transport lasted 5 min) to analyze the serum dosage of the hormones (growth hormone–GH, testosterone–TRT, and estrogen-type estrogen–EST). Subsequently, measurements of growth hormone, testosterone, and estradiol were made in nanogram per deciliter (ng/dL). The levels of these hormones were also measured using the direct chemiluminescence method (i.e., reduction of light as a result of a chemical reaction in a blood sample) with the ADVIA Centaur^®^ XP–SIEMENS (i.e., photomultiplier). The process transforms the light emitted by the chemiluminescence method into electrical impulses and thus the impulses are read in “count” of light per second (i.e., this unit is proportional to the unit of measurement of the hormone levels present in the sample).

## 8. Statistics

The normality of the data was verified using the Kolmogorov–Smirnov and Z-score tests for asymmetry and kurtosis (−1.96–1.96). The estradiol variable had the assumption of normality denied and it was transformed from non-parametric to parametric by the log at the base of 10. Correlations were made using Pearson’s test. In the partial correlations, the effect of the maturational and hormonal variables was statistically controlled. The Schober et al. [34] scale was used for the correlations: Insignificant: r < 0.10; Weak: r = 0.10–0.39; Moderate: r = 0.40–0.69; Strong: r = 0.70–0.89; Very strong: r = 0.90–1.00. Through the median split of the testosterone variable, the sample was categorized into a testosterone group < 100 ng/dL and a testosterone group > 100 ng/dL. Bonferroni correction was applied, and later comparisons were performed using Student’s independent *t*-test. The effect size and the respective 95% confidence intervals were obtained using the Cohen test (d). The magnitude of the effect size followed the classification recommended by Espírito Santo and Daniel [35]: insignificant < 0.19; small 0.20–0.49; mean 0.50–0.79; large 0.80–1.29; very large < 1.30). Linear regressions were performed and the models had the homogeneity tested by the Breush–Pegan test and the assumptions of normality, variance and independence of the data were not denied. Non-linear artificial neural networks of the preceptron type with multiple hidden layers, containing Gausian distributions were programmed. The objective was to determine the prediction of the probability of correct predictions of the maturational and hormonal variables in relation to the neuromuscular variables were used to perform synaptic weight adjustments. Thus, the network activation functions followed the following binary interpretation: (0) False prediction: U < 0, so U is equal to zero; (1) True prediction: U ≤ 0, so U is equal to 1. In addition, for the validation of neural networks, 89.1% of the dataset was used to train the neural networks and 10.9% was used to test the applicability of the programming. During training, the networks were programmed to stop the executions of the seasons when finding the lowest possible error rate for the dataset under analysis, thus an average of 10.000 training seasons was performed. Subsequently, to carry out cross-validation, the database was subdivided into four groups of datasets, 3 with 10 subjects and 1 with 7 subjects, then there was the rotation between training the preceptron network and testing the network, until all groups had gone through both conditions. At the end, the forecasts obtained by the four different groups were added and the average was considered as the final result. For the technical error of anthropometric measurements, the following magnitude was used: Acceptable for skin folds ≤ 5.0%; Acceptable for other anthropometric measurements ≤ 1.0% [36]. All analyses were performed using the R statistical software (version 4.0.1; R Foundation for Statistical Computing^®^, Vienna, Austria), and the significance level of *p* < 0.05 was considered.

## 9. Results

The characterization of the sample shows that the subjects had delayed skeletal maturation, somatic maturation at the prepeak of growth speed and prepubertal sexual maturation (Table 1). The sample showed superiority of lean mass in relation to fat mass in kilograms, and mean levels of growth hormone of 2.27 (ng/dL) and steroid hormones between 20.7 and 40.0 (ng/dL) (estradiol and testosterone, respectively). Regarding anthropometric measurements, technical errors of intra-rater measurements below 3% for skin folds and below 1% for other anthropometric measurements were pointed out. The margin of error pointed out for the sample was 4.83%, being below 5% and the calculated sample power was 0.82. There was no sample loss.

Table 2 shows that testosterone showed a significant relationship with lean mass (kg), estradiol with the performance of SJ and BScD’s and growth hormone showed a relationship with lean mass and the percentage of fat. It was not possible to observe, when statistically controlling for the effect of biological maturation by three different parameters (maturation: skeletal, sexual, and somatic), the correlations of testosterone and growth hormone with lean mass. However, the correlations between estradiol and the performance of SJ and BScD’s remained significant. The same occurred for the correlation of growth hormone with the percentage of body fat.

Table 3 shows that sexual, skeletal, and somatic maturation have significant correlations with lean mass and the performance of ULP and SJ. In addition, it appears that skeletal maturation also showed a significant correlation with ULS.

The linear regression models contained in Table 4 report that biological maturation presented statistical significance as predictors for lean mass, ULP and SJ (W/kg). In addition, skeletal maturation was shown to be significant in helping to predict BScD. Regarding hormone levels, testosterone was significant for the prediction of lean mass, estradiol for the performance of SJ and BScD. Regarding growth hormone, it was only found statistically significant for the prediction of body fat percentage.

The analyses of multilayer neural networks of the preceptron type in Table 5 show that testosterone (ng/dL) has a 49.5% probability of estimating lean mass (kg) and a 49% chance of predicting ULP. Sexual maturation showed 32.5% of correct answers to predict lean mass and 51% of chances in predicting ULP. Skeletal maturation indicated a 35.1% chance of predicting lean mass, 55.4% of predicting ULP and a 34% chance of predicting SJ. While somatic maturation indicated 83.1% of capacity to foresee lean mass, 60% in ULP and 30.3% in SJ. The hormone estradiol (ng/dL) and the growth hormone did not present probabilities of success above 30% for the predetermined predictions.

**Figure 2:**
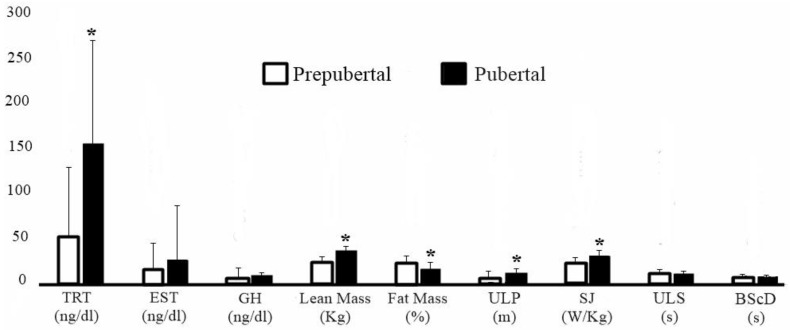
Comparison according to the stage of sexual maturation. * Statistically significant. TRT = Testosterone. EST = Estradiol. GH = Growth Hormone. ULP = Upper limbs power. SJ = Squat Jump. ULS = Upper limb speed. BScD = Body speed with change of direction.

Regarding sexual maturation, 16 subjects were classified as prepubertal (sexual maturation: −2.62 ± 0.54) and 21 as pubertal (sexual maturation: −0.97 ± 0.43). In Figure 2, comparisons between individuals according to maturation stage show that pubertal subjects had higher testosterone levels (effect size: 1.08; 95% CI: [0.36–1.80]; *p* = 0.04); higher concentration of lean mass (effect size: 1.74; 95% CI: [0.95–2.53]; *p* < 0.0001); lower percentage of fat (effect size: 0.72; 95% CI: [0.02–1.41]; *p* = 0.003); and superior performance in ULP (effect size: 1.21; 95% CI: [0.48–1.95]; *p* = 0.0007) and SJ (effect size: 1.29; 95% CI: [0.55–2.03]; *p* = 0.0004), when compared with prepubertal individuals.

When categorizing the sample according to testosterone levels, the two classes discriminated were: testosterone below 100 ng/dL, and testosterone above 100 ng/dL. Thus, the group with testosterone <100 ng/dL had 13 subjects and the testosterone group >100 ng/dL obtained 24 members. The comparisons contained in Table 6 show that the subjects with testosterone >100 ng/dL were statistically higher in relation to ULP and SJ and had a lower body fat percentage compared with individuals with testosterone <100 ng/dL.

## 10. Discussion

The aim of the present study was to relate maturation and hormonal markers to neuromuscular performance, as well as to verify whether young athletes with different levels of testosterone show differences in muscle strength. The main results of the present study were: (1) The hormonal markers showed significant relationships with neuromuscular performance, especially with lower limbs and body morphology. (2) The biological maturation markers were significantly related to body morphology and the performance of ULP, SJ and ULS. (3) The hormonal and biological maturation markers were significant in foreseeing the performance of ULP, SJ, BScD and the levels of lean mass. (4) Subjects categorized as pubescent showed higher concentrations of testosterone and lean mass and better performance of ULP and SJ. (5) Young athletes with TRT > 100 (ng/dL) showed superior performance of ULP and SJ and had a lower percentage of body fat.

The present study found that in young males, hormonal markers point to significant relationships with neuromuscular performance and with levels of lean body mass. Crewther et al. [37] corroborate the findings of the present research when they identified that the levels of steroid hormones in young male weightlifting athletes are related to body mass and show a significant relationship with neuromuscular performance (*p* < 0.05). In a similar perspective, Chin et al. [38] showed in a previous study that the testosterone levels of male subjects indicate significant relationships with the higher levels of upper limb strength and lean body mass. In this sense, Aslam [39] points out that during puberty there is a peak of hormonal levels such as testosterone, estradiol, and growth hormone, which can significantly influence the morphological structure and the acquisition of muscle strength in young people of both genders.

Thus, the findings of the present study also showed significant relationships between biological maturation markers and levels of lean body mass and with the neuromuscular performance of upper and lower limbs. However, the maturation markers did not show significant relationships with the hormonal markers analyzed. Our results differ from the data obtained in the study by Pinto et al. [13], where the authors found significant relationships between skeletal maturation and testosterone in young males (*p* = 0.04). These differences can be justified due to the characteristics of the samples from the two studies (i.e., exact biological age, nutritional factors) and due to the different statistical treatment in relation to the control of laboratory tests. On the other hand, as in the present study, these authors found significant relationships between maturation and neuromuscular performance of the upper and lower limbs of the young people analyzed (*p* < 0.001). In addition, Dantas et al. [40] identified significant relationships between biological maturation and neuromuscular performance of the upper and lower limbs in young Brazilian rowers of both sexes (*p* < 0.05).

The findings of the present study show that the levels of lean mass and the performance of ULP and SJ can be foreshadowed by hormonal and maturation markers. Our group has already demonstrated that through linear regression analyses and artificial neural networks, the biological maturation markers of female subjects show about a 50% probability of predicting the neuromotor performance of upper and lower limbs [14], while hormonal markers discriminated a potential 95% chance of predicting upper limb neuromotor performance [14]. The approach highlights that although environmental factors may significantly interfere with hormonal levels (i.e., food, sleep quality, stress levels), there is a significant association between the stages of puberty and hormonal levels in children and teenagers [41,42,43].

In this context, the results of the present study show that young people with testosterone levels above 100 (ng/dL) are superior in the neuromuscular performance of upper and lower limbs and point to lower body adiposity when compared with their peers with testosterone levels less than 100 (ng/dL). In this perspective, it is emphasized that the findings of the present study indicate that testosterone also increases with growth and development. Therefore, the cut-off value of “100 ng / dL” used in this study is experimental and needs more research to be analyzed in depth. Wirth [44] states that testosterone is related to the acquisition of muscle strength during different periods of human life, including the puberty phase. In this sense, anabolism of skeletal muscle tissue occurs through mechanisms of protein synthesis triggered by testosterone concentrations in the body [45,46].

Moreover, it was observed in the present study that young pubertal boys show higher concentrations of testosterone, greater lean mass and superior neuromuscular performance of upper and lower limbs than the prepubertals. Our results corroborate the study by Distefano et al. [47], where the authors identified that young athletes with advanced puberty had greater knee extension strength when compared with delayed maturation youth. In addition, Pinto et al. [13] found that young people with advanced biological maturation have superior upper and lower limb strength than young people with delayed maturation.

It should be noted that the present study also demonstrated that chronological age is not a safe marker of biological age, when observing that the sample used was in the age group between 10 and 12 years old (age: 11.3 ± 0.96) and the maturation stage typical for this age group is the prepubertal [10,22,41]. However, it is known that environmental factors such as nutrition, quality of sleep, physical and cognitive stimuli interact with the puberty process. Therefore, the stimuli provided can interact with the biological rhythm which can cause the delay or the advancement of puberty in children and adolescents of both sexes [1,10,22,41].

However, despite the relevance of the results, the present study has some limitations: (i) its design is observational, which makes it impossible to establish a cause and effect relationship. (ii) The methodological procedures did not control for the nutritional history of the analyzed subjects, which may have interfered with hormonal concentrations. (iii) Biological maturation markers were measured using predictive formulas based on anthropometry. This fact can lead to a divergence of results in comparison to the analysis of biological maturation using the gold standard (hand and wrist X-ray). (iv) The tests performed may have been influenced by anatomical factors (i.e., length of the upper and lower limbs), biological factors (i.e., level of cerebral excitation and the composition of types of muscle fibers) and an extra level of physical activity (it was not controlled if subjects practiced additional sports).

## 11. Conclusions

Biological maturation and hormone levels may be related to lean mass and neuromuscular performance. Young athletes with higher testosterone levels display less fatty tissue and superior muscle strength in the upper and lower limbs compared with the others. Thus, the present study shows the influence that biological maturation and endocrine markers can have on muscle strength, providing important information for coaches to select sports talents as well as facilitating the application in daily training routines.

## Figures and Tables

**Table 1:** Sample characterization.

Variables	Values
N° Sample (%)	37 (100%)
Bodyweight (kg)	37.3 ± 9.68
Stature (cm)	145.0 ± 7.57
Bodymass index (m²)	17.4 ± 3.67
Fat (%)	22.7 ± 9.21
Lean Mass (kg)	28.4 ± 5.84
Fat Mass (kg)	8.96 ± 5.43
Chronological age	11.3 ± 0.96
Skeletalmaturation (bone age)	10.3 ± 1.21
Sexual maturation	−2.13 ± 0.90
Somaticmaturation (PSG)	−2.61 ± 0.77
Testosterone(ng/dL)	106.6 ± 103.4
Estradiol (ng/dL)	33.2 ± 42.6
Growthhormone(ng/dL)	2.62 ± 2.93
Upperlimbpower (m)	1.70 ± 0.43
SquatJump (W/kg)	25.8 ± 6.95
Upperlimbspeed (s)	12.5 ± 3.67
BScD (s)	8.42 ± 0.71

BScD = Body speed with change of direction

**Table 2:** Correlations of hormonal levels with the study variables.

Variables	TRT (ng/dL)		EST (ng/dL)		GH (ng/dL)	
	r	*p*-Value	r	*p*-Value	r	*p*-Value
Leanmass (kg)	0.40 *	0.02	−0.08	0.6	0.37 *	0.02
Fat (%)	−0.27	0.09	−0.30	0.06	−0.43 *	0.006
Sexualmaturation	0.30	0.06	0.08	0.6	0.28	0.1
Skeletalmaturation	0.27	0.2	0.10	0.5	0.17	0.2
Somaticmaturation	0.27	0.09	0.08	0.6	0.19	0.2
ULP (m)	0.23	0.1	−0.09	0.5	0.09	0.5
SJ (W/kg)	0.28	0.08	−0.33 *	0.04	−0.07	0.6
ULS (s)	−0.06	0.7	0.18	0.2	0.03	0.8
BScD’s (s)	0.07	0.6	0.33 *	0.04	−0.20	0.9
	**Control of the effect of Sexual Maturation**					
	**r**	***p*-Value**	**r**	***p*-Value**	**r**	***p*-Value**
Leanmass (kg)	0.24	0.2	0.23	0.1	0.21	0.2
Fat (%)	0.20	0.2	0.29	0.08	−0.38 *	0.02
ULP (m)	0.08	0.6	0.16	0.3	0.06	0.7
SJ (W/kg)	0.13	0.4	0.49 *	0.002	−0.31	0.05
ULS (s)	0.02	0.8	0.22	0.1	0.12	0.4
COD’s (s)	0.10	0.5	0.34 *	0.04	−0.00	0.9
	**Control of the effect of skeletal maturation**					
	**r**	***p*-Value**	**r**	***p*-Value**	**r**	***p*-Value**
Leanmass (kg)	0.24	0.1	0.27	0.09	0.10	0.5
Fat (%)	0.23	0.1	0.28	0.09	−0.41 *	0.01
ULP (m)	0.10	0.5	0.18	0.2	0.00	0.9
SJ (W/kg)	0.16	0.3	0.49 *	0.002	0.22	0.1
ULS (s)	0.02	0.8	0.24	0.1	0.10	0.5
BScD’s (s)	0.08	0.6	0.33 *	0.04	0.01	0.9
	**Control of the effect of somatic maturation**					
	**r**	***p*-Value**	**r**	***p*-Value**	**r**	***p*-Value**
Leanmass (kg)	0.24	0.1	0.30	0.07	0.17	0.3
Fat (%)	0.25	0.1	0.29	0.08	−0.42 *	0.009
ULP (m)	0.08	0.6	0.18	0.2	0.03	0.8
SJ (W/kg)	0.16	0.3	0.46 *	0.004	0.22	0.1
ULS (s)	0.01	0.9	0.22	0.1	0.09	0.5
BScD’s (s)	0.08	0.6	0.33 *	0.04	0.01	0.9

ULP = Upper limbs power. SJ = Squat Jump. ULS = Upper limb speed. BScD’s = Body speed with change of direction. TRT = Testosterone. EST = Estradiol. GH = Growth Hormone. * Statistically significant.

**Table 3:** Correlations of biological maturation with the study variables.

Variables	Sexual Maturation		Skeletal Maturarion		Somatic Maturation	
	r	*p*-Value	r	*p*-Value	r	*p*-Value
Lean Mass (kg)	0.77 *	<0.0001	0.78 *	<0.0001	0.84 *	<0.0001
Fat (%)	−0.32	0.05	−0.21	0.1	−0.11	0.4
TRT (ng/dL)	0.30	0.06	0.27	0.1	0.27	0.9
EST (ng/dL)	0.08	0.6	0.10	0.5	0.08	0.6
GH (ng/dL)	0.26	0.1	0.17	0.2	0.19	0.2
ULP (cm)	0.52 *	0.0008	0.54 *	0.0004	0.59 *	0.0001
SJ (W/kg)	0.61 *	<0.0001	0.58	0.0001	0.55 *	0.0003
ULS (s)	−0.28	0.08	−0.33 *	0.04	−0.27	0.9
BScD (s)	−0.07	0.6	−0.03	0.8	−0.04	0.8
	**Control of the Effect of TRT (ng/dL)**					
	**r**	***p*-Value**	**r**	***p*-Value**	**r**	***p*-Value**
Lean Mass (kg)	0.74 *	<0.0001	0.76 *	<0.0001	0.83 *	<0.0001
Gordura (%)	0.25	0.1	0.15	0.3	0.04	0.7
ULP (m)	0.48 *	0.002	0.51 *	0.001	0.56 *	0.0003
SJ (W/kg)	0.57 *	0.0002	0.54 *	0.0005	0.51 *	0.001
ULS (s)	−0.27	0.09	−0.33 *	0.04	−0.26	0.1
BScD (s)	−0.09	0.5	−0.05	0.7	−0.06	0.7
	**Control of the Effect of EST (ng/dL)**					
	**r**	***p*-Value**	**r**	***p*-Value**	**r**	***p*-Value**
Lean Mass (kg)	0.78 *	<0.0001	0.80 *	<0.0001	0.85 *	<0.0001
Gordura (%)	−0.31	0.06	0.19	0.2	−0.09	0.5
ULP (m)	0.53 *	0.0007	0.56 *	0.0003	0.60 *	<0.0001
SJ (W/kg)	0.68 *	<0.0001	0.66 *	<0.0001	0.62 *	<0.0001
ULS (s)	−0.30	0.06	−0.36 *	0.02	−0.29	0.07
BScD (s)	−0.10	0.5	−0.07	0.6	−0.07	0.6
	**Control of the Effect of GH (ng/dL)**					
	**r**	***p*-Value**	**r**	***p*-Value**	**r**	***p*-Value**
Lean Mass (kg)	0.78 *	<0.0001	0.78 *	<0.0001	0.85 *	<0.0001
Gordura (%)	−0.23	0.1	0.15	0.3	−0.03	0.8
ULP (m)	0.52 *	0.001	0.54 *	0.0006	0.59 *	0.0001
SJ (W/kg)	0.65 *	<0.0001	0.60 *	<0.0001	0.58 *	0.0001
ULS (s)	−0.30	0.06	−0.34 *	0.03	−0.28	0.08
BScD (s)	0.08	0.6	0.33 *	0.04	0.01	0.9

ULP = Upper limbs power. SJ = Squat Jump. ULS = Upper limb speed. BScD = Body speed with change of direction. TRT = Testosterone. EST = Estradiol. GH = Growth Hormone. * Statistically significant.

**Table 4:** Linear regression of hormonal levels and biological maturation with the study variables.

Variables	Sexual Maturation			Skeletal Maturarion			Somatic Maturation		
	r²	β	*p*	r²	β	*p*	r²	β	*p*
Leanmass (kg)	0.59 *	0.11	0.00	0.61 *	0.15	0.00	0.71 *	0.10	0.00
Fat (%)	0.10	−0.03	0.05	0.04	−0.02	0.1	0.01	−0.00	0.4
TRT (ng/dL)	0.09	0.00	0.06	0.07	0.00	0.1	0.07	0.00	0.09
EST (ng/dL)	0.00	0.00	0.6	0.11	0.00	0.5	0.00	0.00	0.6
GH (ng/dL)	0.07	0.08	0.1	0.03	0.07	0.2	0.03	0.05	0.24
ULP (m)	0.27 *	1.06	0.00	0.29 *	1.38	0.00	0.35 *	0.95	0.00
SJ (W/kg)	0.37 *	0.07	0.00	0.33 *	0.09	0.00	0.30 *	0.05	0.00
ULS (s)	0.08	−0.07	0.08	0.11 *	−0.10	0.04	0.07	−0.05	0.09
BScD (s)	0.00	−0.08	0.6	0.00	−0.05	0.8	0.00	−0.04	0.8
	**TRT (ng/dL)**			**EST (ng/dL)**			**GH (ng/dL)**		
	**r²**	**β**	***p***	**r²**	**β**	***p***	**r²**	**β**	***p***
Leanmass (kg)	0.13 *	6.31	0.02	0.00	−0.65	0.6	0.00	0.03	0.6
Fat (%)	0.07	−3.24	0.09	0.09	−1.48	0.06	0.19 *	−0.13	0.00
Sexual maturation	0.09	34.7	0.06	0.00	3.95	0.6	0.07	0.81	0.1
Skeletalmaturation	0.07	24.6	0.1	0.01	4.17	0.5	0.03	0.43	0.2
Somaticmaturation	0.07	39.7	0.09	0.00	5.20	0.6	0.03	0.75	0.2
ULP (m)	0.05	53.0	0.1	0.00	−9.12	0.5	0.00	0.56	0.5
SJ (W/kg)	0.08	4.26	0.08	0.11 *	−2.11	0.04	0.00	−0.03	0.6
ULS (s)	0.00	−1.90	0.7	0.03	2.36	0.2	0.00	0.02	0.8
BScD (s)	0.00	10.2	0.6	0.11 *	19.8	0.04	0.00	−0.07	0.9

ULP = Upper limbs power. SJ = Squat Jump. ULS = Upper limb speed. BScD = Body speed with change of direction. TRT = Testosterone. EST = Estradiol. GH = Growth Hormone. * Statistically significant.

**Table 5:** Analysis of perceptron neural networks for forecasting possibilities.

Variables	TRT (ng/dL)			EST (ng/dL)			GH (ng/dL)		
	U	%E	|P%	U	%E	|P%	U	%E	|P%
Leanmass (kg)	1 *	50.5	49.5	0	77.4	22.6	1 *	77.9	22.05
Fat (%)	1 *	83.0	17.0	1 *	94.6	5.34	0	85.0	15.0
Sexual maturation	0	80.3	19.7	0	99.9	0.06	1 *	99.9	0.02
Skeletalmaturation	1 *	90.0	10.0	0	95.9	4.10	0	97.3	2.62
Somaticmaturation	1 *	87.4	12.6	1 *	99.5	0.46	1 *	99.1	0.84
ULP (m)	1 *	51.0	49.0	0	89.7	10.22	0	97.3	1.65
SJ (W/kg)	1 *	78.6	23.4	1 *	90.5	9.42	1 *	98.5	1.44
ULS (s)	1 *	75.4	24.6	1 *	73.7	26.3	1 *	98.5	1.45
BScD (s)	1 *	80.4	19.6	1 *	97.8	2.16	1 *	94.9	5.09
	**Sexual maturarion**			**Skeletalmaturation**			**Somaticmaturation**		
	**U**	**%E**	**|P%**	**U**	**%E**	**|P%**	**U**	**%E**	**|P%**
Leanmass (kg)	0	67.5	32.5	1 *	64.9	35.1	1 *	16.9	83.1
Fat (%)	1 *	74.8	25.2	1 *	79.3	20.7	1 *	84.8	15.2
TRT (ng/dL)	1 *	72.6	27.4	1 *	80.7	19.3	0	85.0	15.0
EST (ng/dL)	0	85.7	14.3	1 *	86.6	13.4	1 *	87.5	12.5
GH (ng/dL)	1 *	94.2	5.73	1 *	95.0	5.00	1 *	94.8	5.2
ULP (m)	1 *	48.3	51.7	1 *	44.6	55.4	1 *	40.0	60.0
SJ (W/kg)	0	70.3	29.7	1 *	66.0	34.0	0	69.7	30.3
ULS (s)	0	97.7	2.30	1 *	96.6	3.35	1 *	98.5	1.5
BScD (s)	0	99.1	0.90	1 *	98.2	1.75	1 *	97.0	3.00

ULP = Upper limbs power. SJ = Squat Jump. ULS = Upper limb speed. BScD = Body speed with change of direction. TRT = Testosterone. EST = Estradiol. GH = Growth Hormone. U = Domain of the Gaussian function of binary activation of the neural network. % E = Percentage of total neural network learning error. | P% = Percentage of the probability of the forecast is correct. * = True forecast.

**Table 6:** Comparisons between subjects with testosterone levels < 100 (ng/dL) and > 100 (ng/dL).

Variables	TRT < 100 (ng/dL)	TRT > 100 (ng/dL)	ES	IC (95%)	*p*
Sexual maturation	−2.13 ± 0.90	−1.49 ± 0.95	−0.69	[−1.41–0.01]	0.05
Skeletal maturation	10.3 ± 1.21	11.0 ± 1.07	−0.59	[−1.30–0.12]	0.08
Somaticmaturation	−2.61 ± 0.77	−2.18 ± 0.65	−0.58	[−1.29–0.13]	0.08
Fat (%)	22.7 ± 9.21	15.6 ± 8.06 *	0.80	[0.07–1.52]	0.02
Leanmass (kg)	28.4 ± 5.84	31.9 ± 6.45	−0.58	[−1.29–0.13]	0.1
TRT (ng/dL)	40.0 ± 26.4	221.7 ± 91.8 *	3.13	[2.11–4.15]	<0.0001
EST (ng/dL)	20.7 ± 55.1	20.7 ± 22.8	0.00	[−0.69–0.69]	0.9
GH (ng/dL)	2.27 ± 3.10	3.26 ± 2.58	−0.36	[−1.03–0.36]	0.3
ULP (m)	1.70 ± 0.43	1.85 ± 0.54 *	0.28	[−0.41–0.98]	0.04
SJ (W/kg)	25.8 ± 6.95	30.2 ± 7.54 *	0.60	[−1.32–0.10]	0.03
ULS (s)	12.5 ± 3.67	12.3 ± 3.81	0.05	[−0.64–0.75]	0.8
BScD(s)	8.42 ± 0.71	8.64 ± 0.88	−0.29	[−0.99–0.41]	0.4

ULP = Upper limbs power. SJ = Squat Jump. ULS = Upper limb speed. BScD = Body speed with change of direction. TRT = Testosterone. EST = Estradiol. GH = Growth Hormone. * Statistically significant. ES—effect size
